# Identification and Verification of Feature Biomarkers Associated With Immune Cells in Dilated Cardiomyopathy by Bioinformatics Analysis

**DOI:** 10.3389/fgene.2022.874544

**Published:** 2022-05-12

**Authors:** Tingfang Zhu, Mingjie Wang, Jinwei Quan, Zunhui Du, Qiheng Li, Yuan Xie, Menglu Lin, Cathy Xu, Yucai Xie

**Affiliations:** ^1^ Department of Cardiovascular Medicine, Ruijin Hospital, Shanghai Jiao Tong University School of Medicine, Shanghai, China; ^2^ Johns Hopkins University, Baltimore, MD, United States

**Keywords:** dilated cardiomyopathy, immune infiltration, diagnosis model, biomarkers, logistic regression

## Abstract

**Objective:** To explore immune-related feature genes in patients with dilated cardiomyopathy (DCM).

**Methods:** Expression profiles from three datasets (GSE1145, GSE21610 and GSE21819) of human cardiac tissues of DCM and healthy controls were downloaded from the GEO database. After data preprocessing, differentially expressed genes (DEGs) were identified by the ‘limma’ package in R software. Gene Ontology (GO) and Kyoto Encyclopedia of Genes and Genomes (KEGG) pathway enrichment analyses were then performed to identify biological functions of the DEGs. The compositional patterns of stromal and immune cells were estimated using xCell. Hub genes and functional modules were identified based on protein-protein interaction (PPI) network analysis by STRING webtool and Cytoscape application. Correlation analysis was performed between immune cell subtypes and hub genes. Hub genes with |correlation coefficient| > 0.5 and *p* value <0.05 were selected as feature biomarkers. A logistic regression model was constructed based on the selected biomarkers and validated in datasets GSE5406 and GSE57338.

**Results:** A total of 1,005 DEGs were identified. Functional enrichment analyses indicated that extracellular matrix remodeling and immune and inflammation disorder played important roles in the pathogenesis of DCM. Immune cells, including CD8^+^ T-cells, macrophages M1 and Th1 cells, were proved to be significantly changed in DCM patients by immune cell infiltration analysis. In the PPI network analysis, STAT3, IL6, CCL2, PIK3R1, ESR1, CCL5, IL17A, TLR2, BUB1B and MYC were identified as hub genes, among which CCL2, CCL5 and TLR2 were further screened as feature biomarkers by using hub genes and immune cells correlation analysis. A diagnosis model was successfully constructed by using the three biomarkers with area under the curve (AUC) scores 0.981, 0.867 and 0.946 in merged dataset, GSE5406 and GSE57338, respectively.

**Conclusion:** The present study identified three immune-related genes as diagnostic biomarkers for DCM, providing a novel perspective of immune and inflammatory response for the exploration of DCM molecular mechanisms.

## Introduction

Dilated cardiomyopathy (DCM) is defined as left or both ventricles enlargement and contraction impairment in the absence of abnormal loading conditions or coronary disease. The estimated prevalence of DCM was >1:250 of the population (Hershberger et al., 2013). It accounts for a considerable portion of heart failure (HF) (Reichart et al., 2019) and is the leading cause of heart transplantation (Weintraub et al., 2017). DCM results from a diverse range of etiologies including genetic alteration, viral infection, drug and alcohol with a heterogeneous pathophysiological mechanism.(Felker et al., 2000).

Immune and inflammatory response plays an important role in cardiovascular disease, such as myocardial infarction (Kologrivova et al., 2021), atrial fibrillation (Li et al., 2021). As for DCM, myocardial damage, whether from a genetic or environmental etiology, triggers inflammation and recruits immune cells to the heart. Regional inflammation causes tissue fibrosis, which stiffens the heart and promotes the progression to dilation and HF (Schultheiss et al., 2019). Myocardial inflammation was related to poor long-term outcome for DCM (Nakayama et al., 2017). Immune cells, especially T lymphocytes and macrophages, promote myocardial inflammation and contribute to ventricular remodeling (Comarmond and Cacoub, 2017; Jain et al., 2021). For example, it is been reported that polarization of macrophages towards M2 was associated with ventricular remodeling and poor long-term prognosis in DCM (Nakayama et al., 2017). In addition, a significant increase of the number of Th1 and Th17 cells was observed while the number of Treg cells decreased in DCM patients (Wei et al., 2017; Liu et al., 2021). Infiltrated immune cells release cytokines and chemokines, such as TGF-β1, IL-1β, and TNF, promoting collagen deposition, fibrosis and cardiac remodeling (Schultheiss et al., 2019). Microarray profiling research has also shown that the expression of some immune-related genes in left ventricle of DCM was dysregulated, such as IL-6, CXCL10, TLR3 (Qiao et al., 2017). Based on the potential relationship between inflammation and DCM, some immunological therapies have been reported, including immunosuppressants, (Parrillo et al., 1989; Frustaci et al., 2009), immunoadsorption, (Bian et al., 2021), IL-1 inhibitors, (Van Tassell et al., 2017; De Luca et al., 2018). However, these immune-based therapies are either unsatisfactory or not fully confirmed by large randomized, multi-center research. It is necessary to better understand DCM pathogenetic mechanism.

**FIGURE 1:**
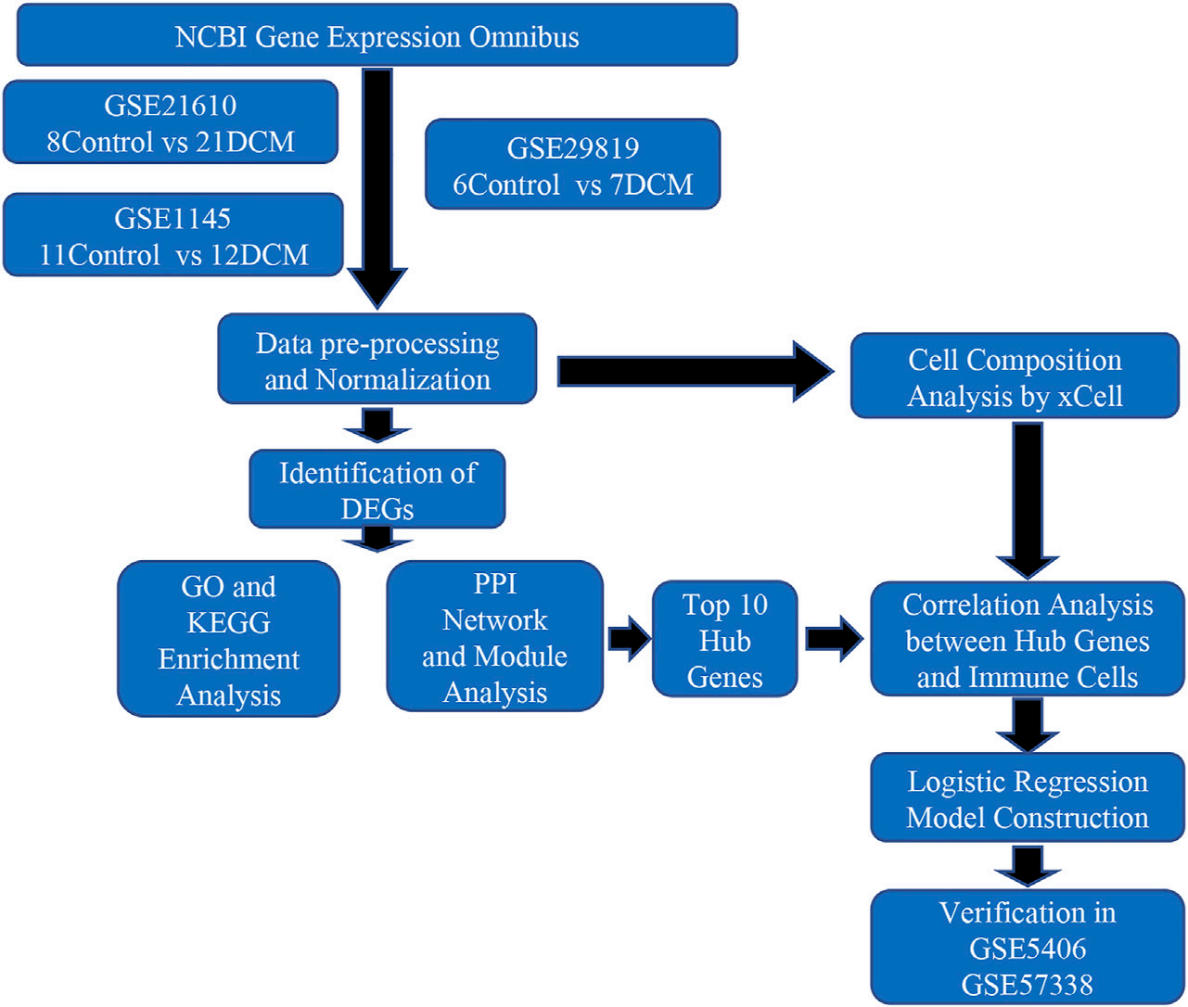
Flowchart of the study.

As shown in Figure 1, we integrated several GEO datasets. Through systematically bioinformatics analyses, we identified differentially expressed genes (DEGs) between DCM and healthy cardiac samples and explored the potential pathological mechanism of DCM by functional enrichment analysis, immune cell infiltration analysis and protein-protein network analysis. Moreover, we constructed a three-gene diagnostic model via logistic regression analysis. Finally, we confirmed the validity of the diagnostic model in another two datasets GSE406 and GSE57338. This article is the first to explore the pathogenesis of DCM from the perspective of immunity and inflammation with bioinformatics, and we hope our analyses will provide potential targets for future in-depth research.

## Materials and Methods

### GEO Datasets

The DCM RNA expression datasets were collected from the online GEO database (http://www.ncbi.nlm.nih.gov/geo/). The keywords “dilated cardiomyopathy”, “Homo sapiens”, and “expression profiling by array” were used on the initial search, and 184 DCM related studies were found. Then the following criteria were used to further screen datasets: 1) The study includes DCM case vs healthy control; 2) tissue samples obtained from left ventricle; 3) sample size was bigger than 10. Three datasets qualified for the above criteria and performed on the same platform were combined for analysis. Another two datasets derived from other platforms were used as validating datasets. The processed data of GSE1145 (platform: GPL570, including 11samples of control and 12 samples of DCM), GSE21610 (Schwientek et al., 2010) (platform: GPL570, including eight samples of control and 21 samples of DCM), GSE29819 (Gaertner et al., 2012) (platform: GPL570, including six samples of control and seven samples of DCM), GSE5406 (Hannenhalli et al., 2006) (platform: GPL96, including 16 samples of control and 86 samples of DCM) GSE57338 (Liu et al., 2015) (platform: GPL11532, including 132 samples of control and 82 samples of DCM) were downloaded as expression matrix with R package ‘GEOquery’.(Davis and Meltzer, 2007). The mRNA expression profiles of controls and targeted patients were extracted and were performed log2 transformation before further analysis (Only if they have not be log2 transformed).

### Data Preprocessing and DEGs Identification

The ‘limma’ (Ritchie et al., 2015) and ‘sva’ (Parker et al., 2014) packages in R software (R version 4.0.3) were used to correct intra- and inter-batch effect. Prcomp function was used to perform principal component analysis (PCA). Next, probes annotations were performed. Probes annotated to >1 gene were removed. For multiple probes annotated to the same gene, the first one appeared retained. Finally, the ‘limma’ package was also used for DEGs identification between different groups with cut-off values of adjusted *p*-value < 0.05 and |fold change| ≥ 1.5. In addition, DEGs were also identified by applying robust rank aggregation (RRA) algorithm (Kolde et al., 2012) with the same criteria. Venn diagrams (http://bioinformatics.psb.ugent.be/webtools/Venn/) were used to summarize the overlapping DEGs between ‘sva’ and RRA algorithms. Boxplot function, ‘ggplot2’ and ‘pheatmap’ packages were then used to plot gene expression boxplot, volcano plot and heatmap.

### Functional Enrichment Analysis

To further analyze the functions of DEGs, the R package ‘clusterProfiler’ (Yu et al., 2012) was used to perform Gene Ontology (GO) and Kyoto Encyclopedia of Genes and Genomes (KEGG) enrichment analysis. The cut-off values for GO and KEGG were set as *p* < 0.05. The ‘enrichplot’ package was used to draw dot plots for the results of functional enrichment analysis.

### Immune Cell Infiltration Analysis

xCell (Aran et al., 2017) is a new gene signature-based method to estimate the content of immune and stromal cells. It was validated using extensive in-silico simulations and cytometry immunophenotyping. XCell package was applied to normalized merged data to portray the cellular heterogeneity landscape of left ventricular expression profiles. We compared the cell distribution differences between the two groups through *t*-test, and the cutoff value was set as *p* < 0.05. The results were significantly different between the two groups, categorized based on their traits into three categories: “lymphoid and myeloid cells”, “stem cells” and “stromal cells and others.” These were visualized by using ‘ggplot2’ and ‘ggpubr’ packages. Correlation analyses of immune cell subtypes and hub genes were performed with ‘psych’ and ‘corrplot’ packages. Pearson correlation coefficient was used to assess the strength of correlation. Hub genes whose absolute correlation coefficient with immune cells >0.5 and *p*-value < 0.05 are selected to further study.

### Protein-Protein Interaction Network and Gene Module Identification

Search Tool for the Retrieval of Interacting Genes (STRING, https://string-db.org) is a webtool that provide validated and predicted information of PPIs (Szklarczyk et al., 2019). The list of DEGs was uploaded into STRING website to detect significant protein interactions with minimum interaction score >0.7. The network was then exported and visualized by Cytoscape 3.7.1 software (Cline et al., 2007). The CytoHubba plugin (Chin et al., 2014) was used to calculate hub genes with a high degree. The results were directly visualized by Cytoscape. Additionally, the MCODE plugin (Bader and Hogue, 2003) was used to identify highly interconnected clusters with the cutoff parameters set as follows: degree cutoff = 2, node score cutoff = 0.2, k-Core = 2, Max. Depth = 100. The results were further screened with criteria set as MCODE score >4 and nodes number >5. Gene ontology biological process enrichment analysis was performed on the significant modules.

### Hub Genes Verification and Diagnosis Model Construction

Hub genes with correlation coefficient >0.5 and *p* < 0.05 with immune cells were selected. The expression profiles of the selected biomarkers were visualized in boxplot and validated in another two datasets GSE5406 and GSE57338. Then, a diagnosis model combining the selected biomarkers was constructed in the merged dataset by logistic regression using ‘glm’ function and verified in GSE5406 and GSE57338. Receiver-operating characteristic (ROC) curves were used to assess the discrimination ability of the key genes and the diagnosis model.

## Results

### Data Preprocessing and DEGs Identification

**FIGURE 2:**
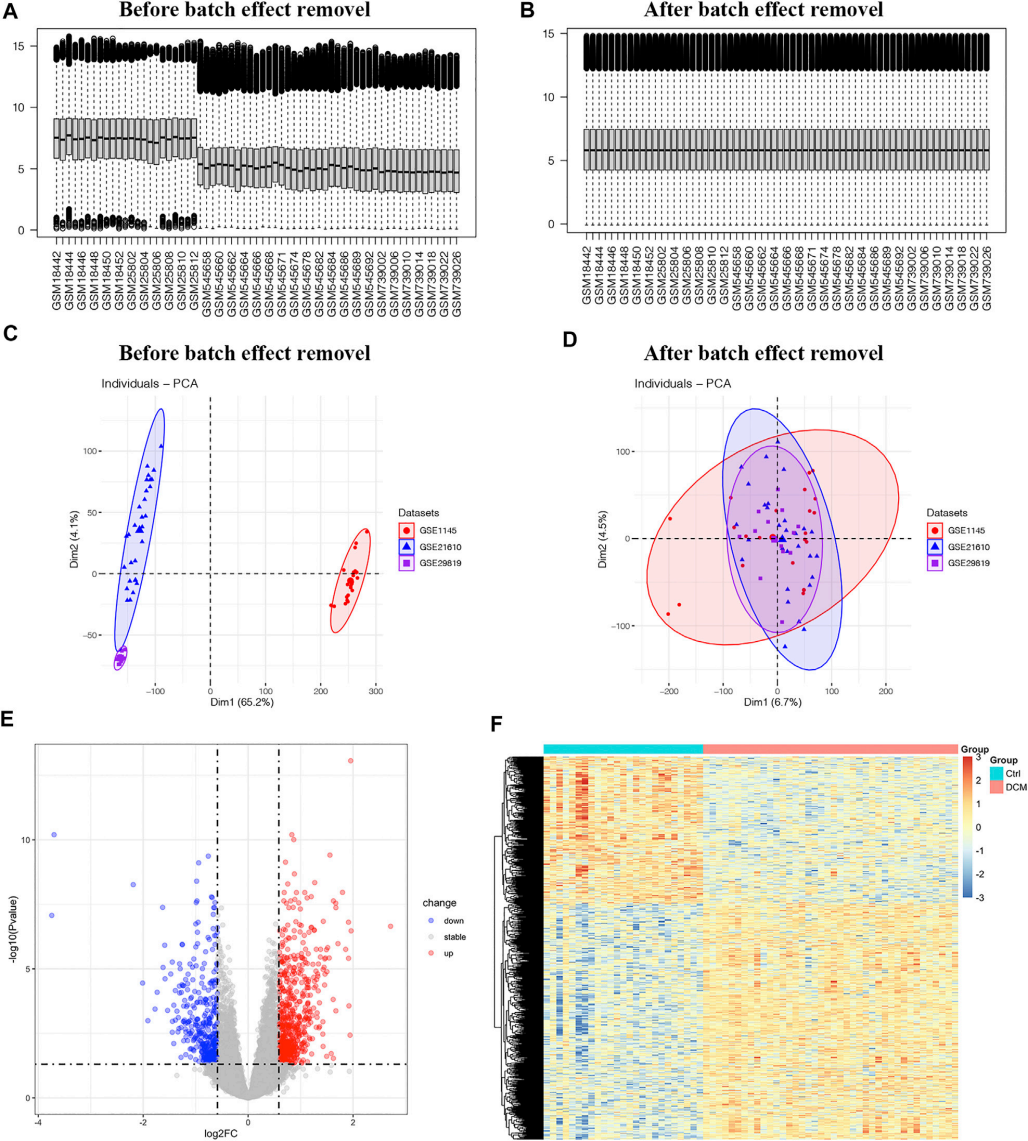
The differentially expressed genes in merged dataset **(A,B)** The boxplot of merged dataset before and after normalization **(C,D)** Two-dimensional principal component analysis cluster plot before and after normalization **(E)** Volcano plot of 1,005 DEGs **(F)** Heatmap of all DEGs.

Expression profiles of healthy controls and DCM patients from GSE1145, GSE21610 and GSE21869 were combined into a merged dataset. The merged dataset contains 25 healthy controls and 40 DCM patients. As shown in Figures 2A,C, the merged dataset had strong batch effect. After normalization, it was effectively removed (Figures 2B,D). Then in the differential expression analysis, 1,005 DEGs were recognized in the merged dataset, which includes 385 down-regulated genes and 620 up-regulated genes by integration method of ‘sva’ (Figure 2E). 179 DEGs including 101 up-regulated genes and 78 down-regulated genes were identified by integration method of RRA. Venn diagrams depict DEGs across two different integration methods (Supplementary Figure S1). DEGs obtained by ‘sva’ method were used in the following analyses. All of the DEGs were displayed in heatmap (Figure 2F).

### Functional Enrichment Analysis

**FIGURE 3:**
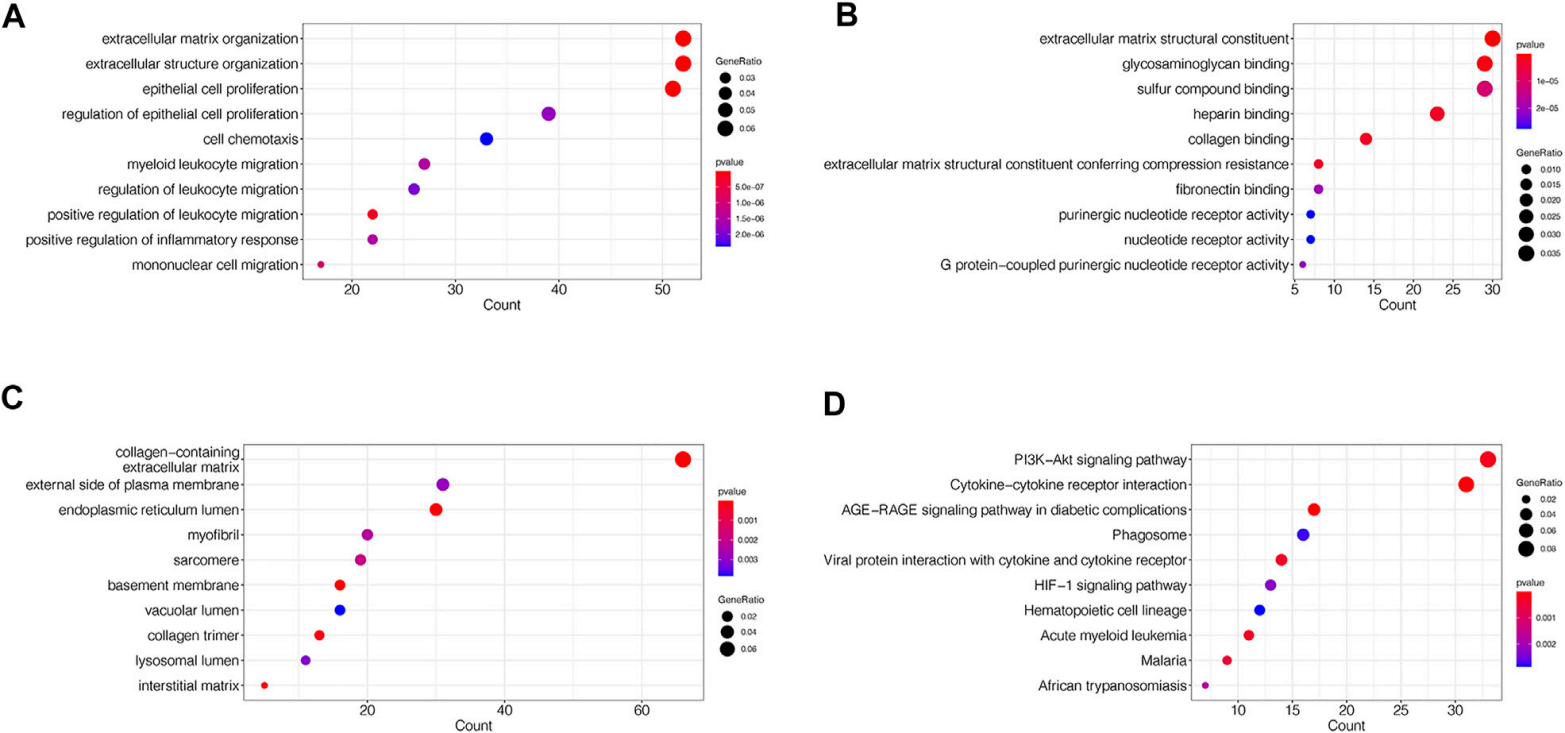
GO and KEGG enrichment analyses of DEGs in merged dataset **(A)** The dot plots of the top 10 enriched GO BP terms **(B)** The dot plots of the top 10 enriched GO MF terms **(C)** The dot plots of the top 10 enriched GO CC terms **(D)** The dot plots of the top 10 enriched KEGG terms.

To assess the functions of DEGs, enrichment analyses of GO and KEGG were performed. The categories of GO analysis include biological process (BP), cellular component (CC) and molecular function (MF). The leading 10 enriched terms of each GO categories and KEGG with *p* value <0.05 were visualized in Figure 3.

The mainly enriched BP terms included epithelial cell proliferation and its regulation, extracellular matrix and structure organization, cell chemotaxis, leukocyte migration and regulation, and inflammatory response (Figure 3A). The mainly enriched MF terms were extracellular matrix structural constituent, glycosaminoglycan, collagen and fibronectin binding, G protein-coupled purinergic nucleotide receptor activity (Figure 3B). The mainly enriched CC terms contained collagen-containing extracellular matrix, endoplasmic reticulum lumen, external side of plasma membrane, myofibril, sarcomere, basement membrane, and collagen trimer (Figure 3C). In whole, GO results indicated that the gene function of DEGs mainly associated with both extracellular structure reorganization and fibrosis, immune, and inflammatory abnormalities.

In KEGG pathway enrichment analysis, DEGs mainly enriched in PI3K-Akt signaling pathway, Cytokine-cytokine receptor interaction, AGE-RAGE signaling pathway in diabetic complications, Phagosome, viral protein interaction with cytokine and cytokine receptor, and HIF-1 signaling pathway (Figure 3D).

### Immune Cell Infiltration Analysis

**FIGURE 4:**
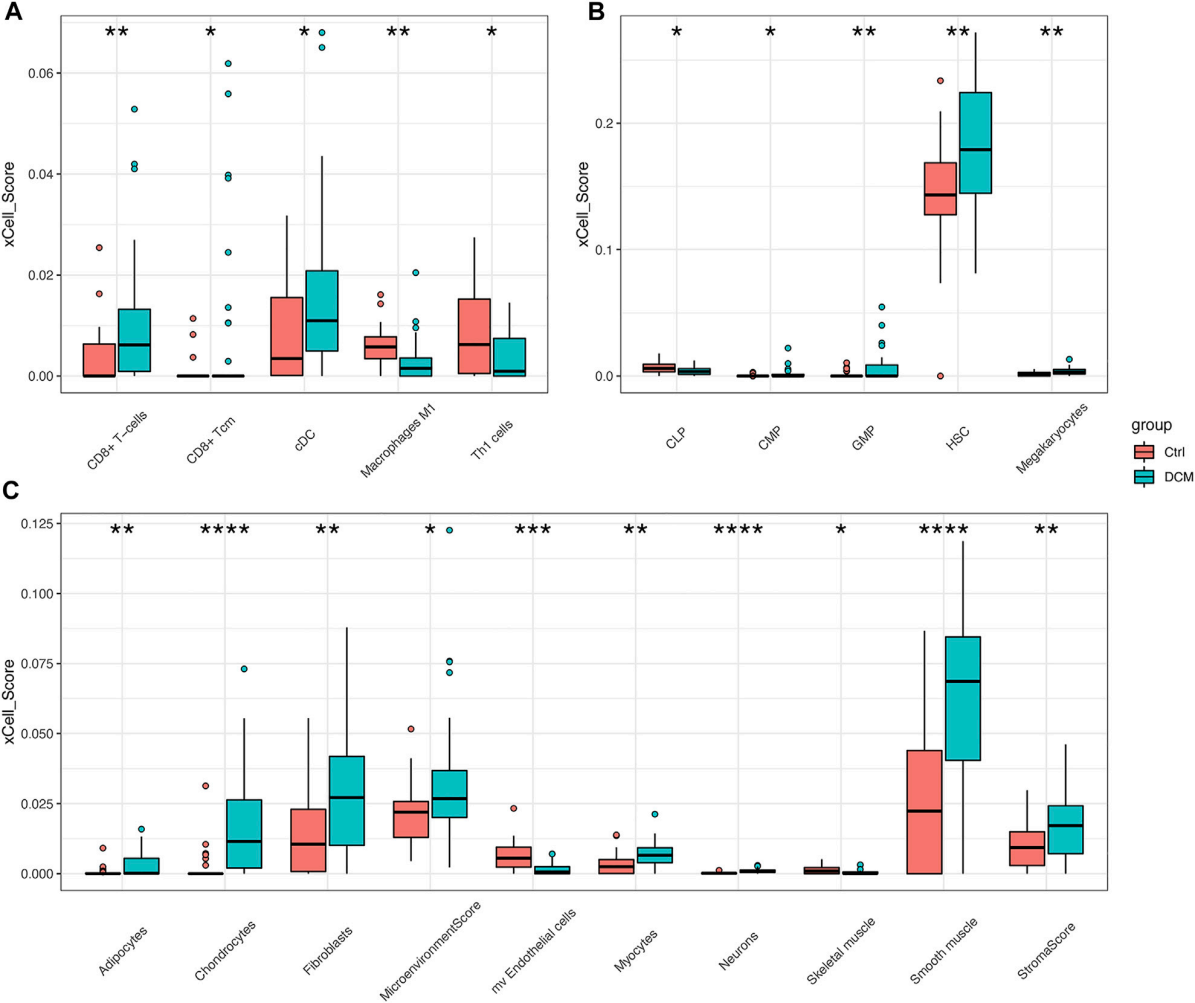
xCell scores of immune and stromal cells between DCM and control heart tissues in merged dataset **(A–C)** Boxplots of “lymphoid and myeloid cells”, “stem cells”, “stromal cells and others” respectively. *p < 0.05, **p < 0.01, ***p < 0.001, ****p < 0.0001.

xCell was used to estimate the cell composition heterogeneity of left ventricle between DCM and controls. As shown in Figure 4, 18 cell types were significantly changed in DCM cardiac tissue compared to control samples, among which the scores of CD8^+^ T-cells, cDC, adipocytes, fibroblasts, and smooth muscle in DCM were significantly increased, while the scores of macrophages M1, Th1 cells were significantly decreased.

**FIGURE 5:**
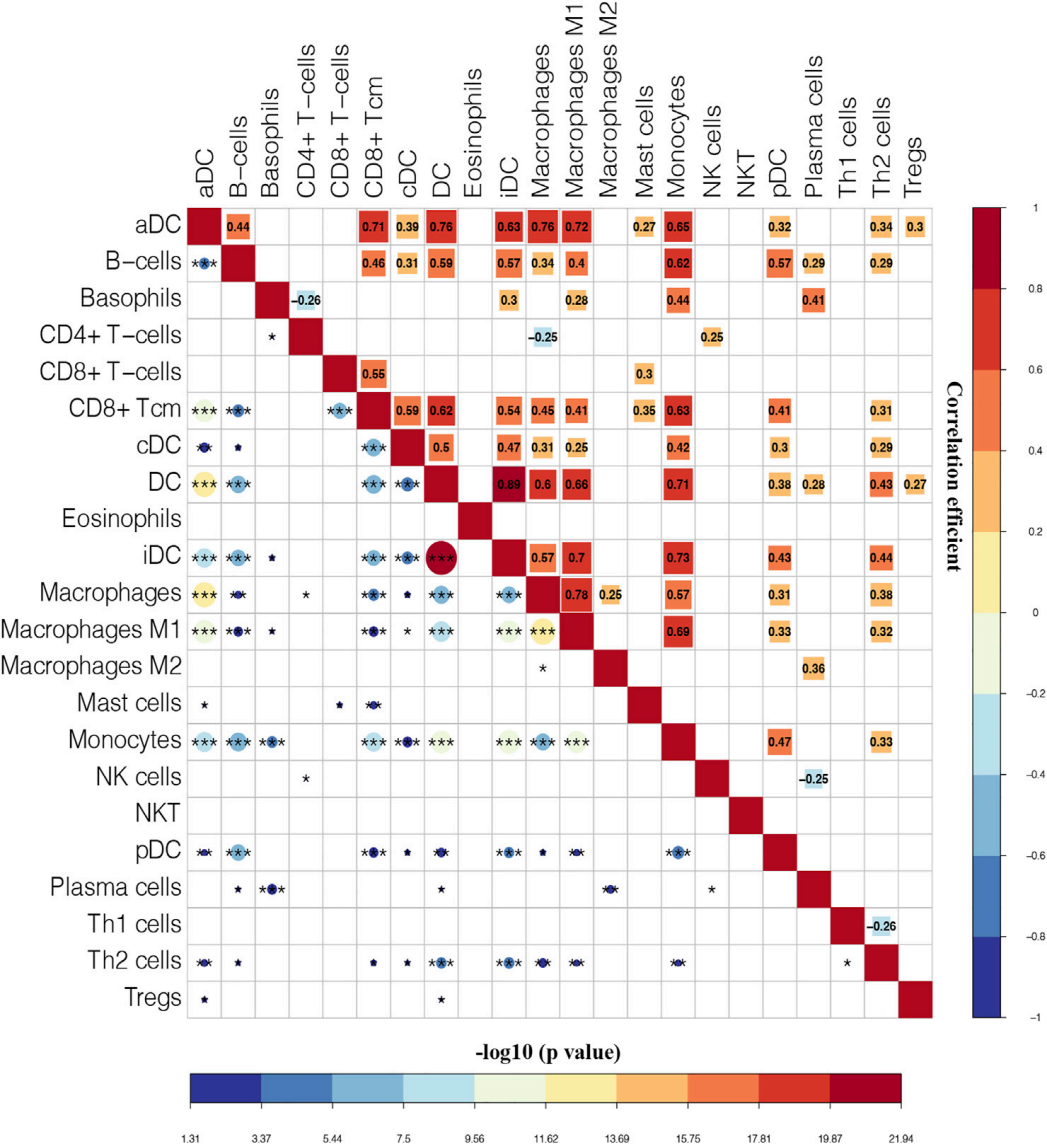
Correlation matrix of immune cells. Blue squares represent negative correlation, and red squares represent positive correlation. The size of the square in the upper right is positively correlated with the correlation coefficient. The size of the circle in the lower left is positively correlated with the significance of the correlation. *p < 0.05, **p < 0.01, ***p < 0.001.

Moreover, the correlation among immune cells were calculated by using the Pearson’s correlation coefficients. As shown in Figure 5, iDC has the highest positive correlation with DC (Pearson’s coefficient = 0.89). The correlation between macrophages and macrophages M1 was the second strongest positive (Pearson’s coefficient = 0.78). Additionally, aDC, DC, CD8^+^ Tcm, macrophages, monocytes, and macrophages M1 had strong correlation coefficient with most of the remaining immune cells.

### PPI Network and Gene Module Identification

**FIGURE 6:**
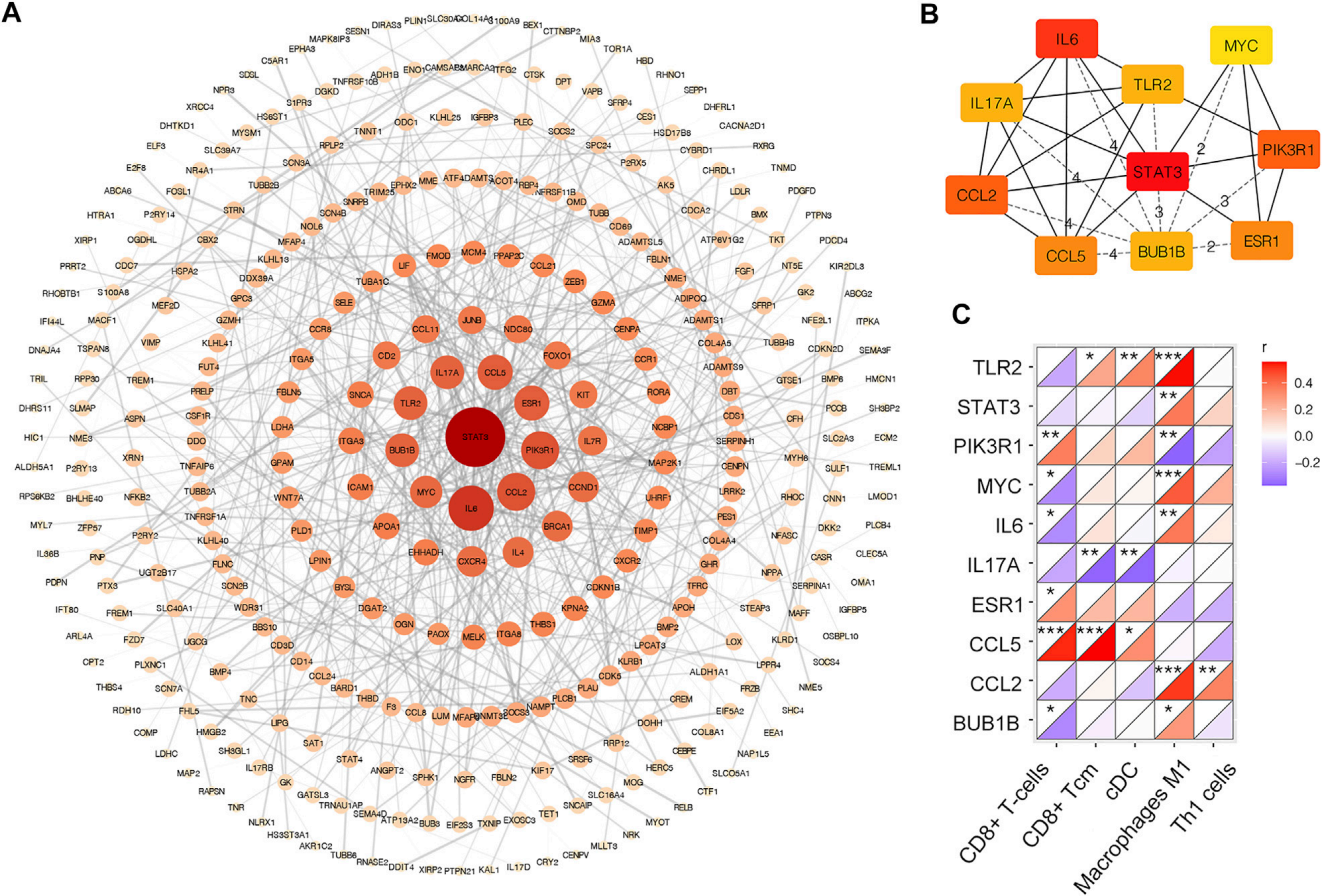
PPI network of DEGs in merged dataset **(A)** PPI network of DEGs. The color depth and shape size of the dots are positively correlated with degree **(B)** Top 10 hub genes in the network **(C)** Correlation matrix of xCell scores of significantly changed immune cell subtype with top 10 hub genes. *p < 0.05, **p < 0.01, ***p < 0.001.

As shown in Figure 6A, there were 633 edges among 350 proteins in the PPI network. The top 10 genes with the highest degree in the above network were selected as the hub genes. These are STAT3, IL6, CCL2, PIK3R1, ESR1, CCL5, IL17A, TLR2, BUB1B, and MYC (Figure 6B). The expression of the top 10 hub genes across all datasets was displayed in supplementary material. Then, the correlation coefficients between 10 hub genes and significantly changed immune cells were calculated. As shown in Figure 6C, CD8^+^ T-cells and macrophages M1 had significant correlation with most of the hub genes. Moreover, with the screening rules as |Pearson’s coefficient| > 0.5 and *p* < 0.05, we obtained three immune-related hub genes with potential diagnostic value: TLR2, CCL2 and CCL5.

Additionally, the densely connected modules were identified by using MCODE plug-in. Nine modules were obtained from the PPI network (Supplementary Figure S2). The most enriched GO BP terms of each module were listed in Table 1. Module_1 was enriched in negative regulation of transcription from RNA polymerase II promoter. Module_2 was enriched in sister chromatid cohesion. Enriched BP terms of Module_3 and four were associated with metabolism. Module_5 was enriched in proteolysis. Module_6 and eight were enriched in extracellular matrix organization. Module_9 was enriched in immune response.

**TABLE 1:** Gene ontology biological process enrichment analysis.

Module	Term	Count	*p* Value
1	negative regulation of transcription from RNA polymerase II promoter	6	7.94E-07
2	sister chromatid cohesion	4	3.98E-06
3	metabolic process	2	3.98E-02
4	keratan sulfate catabolic process	5	1.58E-13
5	proteolysis	4	4.00E-04
6	extracellular matrix organization	3	7.94E-04
8	extracellular matrix organization	5	2.00E-08
9	immune response	6	1.00E-08

### Hub Genes Verification and Diagnosis Model Construction

**FIGURE 7:**
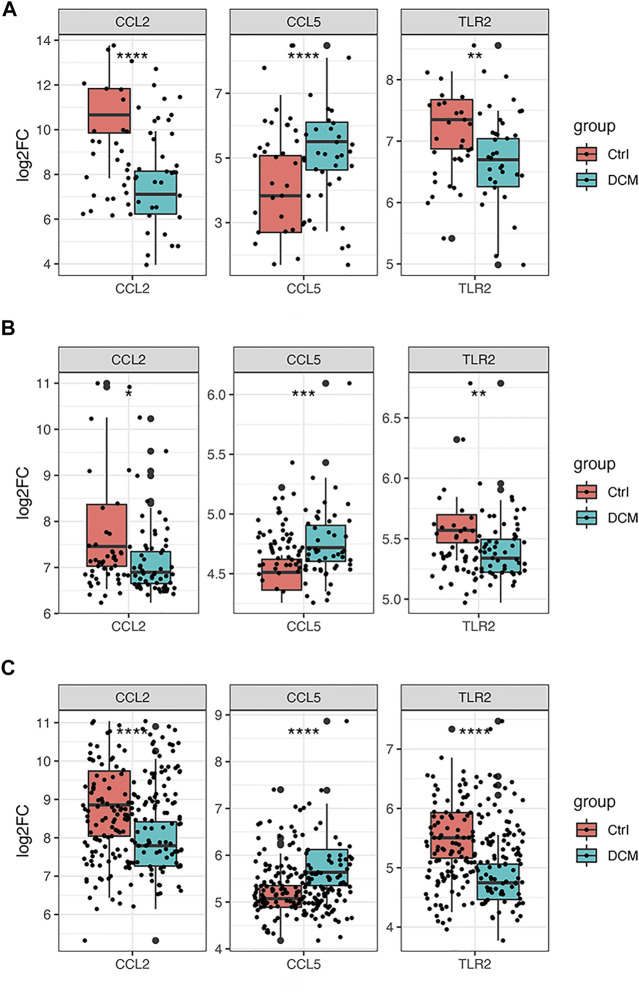
Gene expression of the three selected hub genes **(A)** Boxplot of the three selected hub genes in merged dataset **(B)** Boxplot of the three selected hub genes in GSE5406 **(C)** Boxplot of the three selected hub genes in GSE57338. *p < 0.05, **p < 0.01, ***p < 0.001 ****p < 0.0001.

**FIGURE 8:**
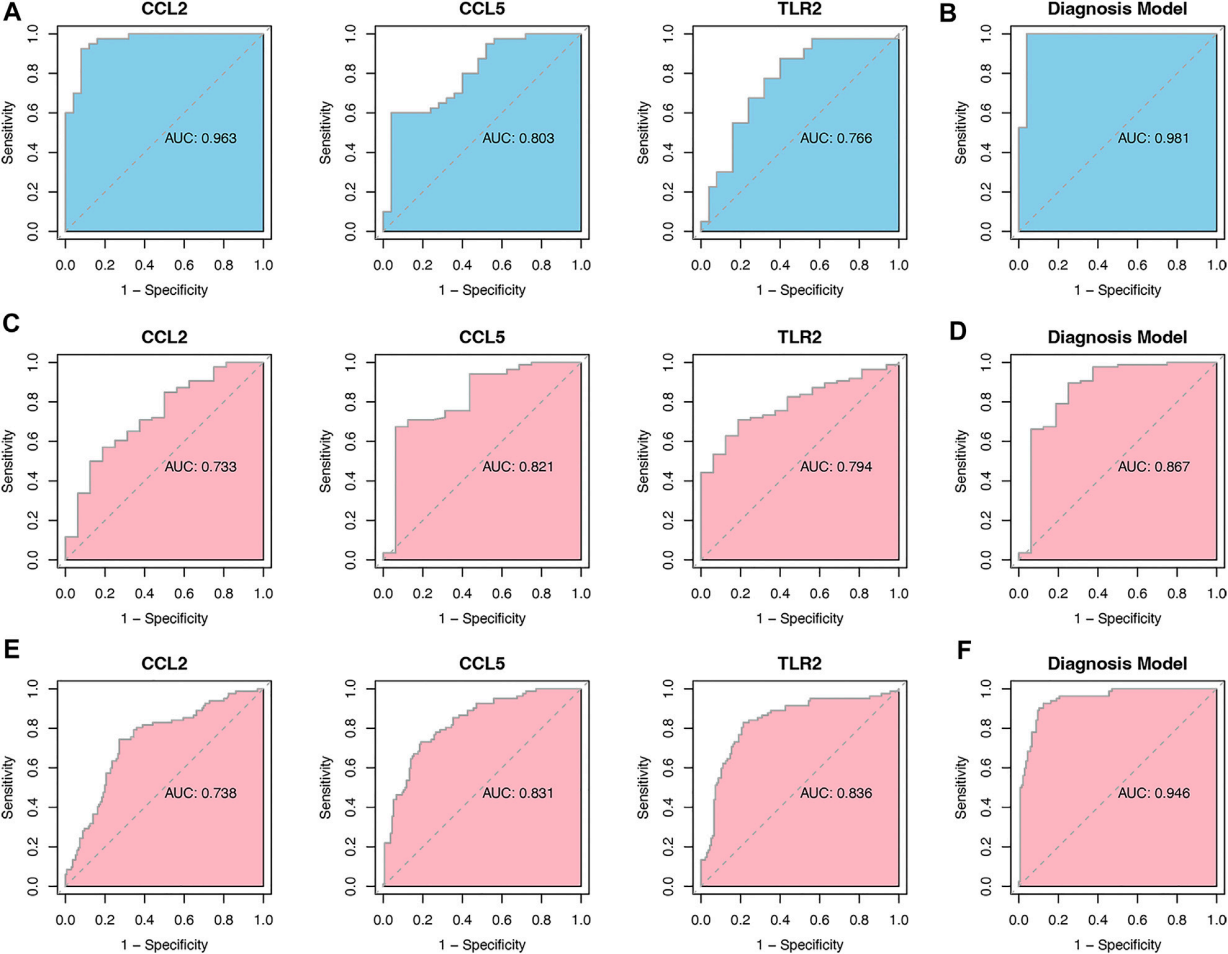
Analysis of the disease predictive abilities of the three selected hub genes **(A)** ROC curve analysis of three selected hub genes in merged dataset **(B)** ROC curve analysis of diagnosis model using the three selected hub genes in merged dataset **(C)** ROC curve analysis of three selected hub genes in GSE5406 **(D)** ROC curve analysis of diagnosis model using the three selected hub genes in GSE5406 **(E)** ROC curve analysis of three selected hub genes in GSE57338 **(F)** ROC curve analysis of diagnosis model using the three selected hub genes in GSE57338.

As it is shown in Figure 7A, CCL2 and TLR2 were significantly downregulated, while CCL5 was significantly upregulated in the merged dataset and verified in another two datasets GSE5406 and GSE57338 (Figures 7B,C). Next, ROC curve analysis was performed to verify the diagnostic value of the selected biomarkers. In the merged dataset, the ROC curves of the three biomarkers revealed high diagnostic value for DCM (Figure 8A). The AUC (area under the curve) scores of CCL2, CCL5, TLR2 were 0.963, 0.803, 0.766 respectively. In verification dataset GSE5406, the AUC scores of CCL2, CCL5, TLR2 were 0.733, 0.821, 0.794, respectively (Figure 8C). In verification dataset GSE57338, the AUC scores of CCL2, CCL5, TLR2 were 0.738, 0.831, 0.836, respectively (Figure 8E). Finally, we used the three biomarkers to construct a diagnosis model by logistic regression and visualized in ROC curves. The AUC score of the diagnosis model was 0.981, 0.867 and 0.946 in the merged dataset, GSE5406 and GSE57338, respectively (Figures 8B,D,F).

## Discussion

DCM, a heterogeneous disease, is the major cause of heart failure and heart transplantation worldwide. Both genetic mutation and many different environmental changes can cause cardiomyocyte damage or death and may trigger myocardial inflammation in both directions, further promoting the progression of cardiomyopathy (Whelan et al., 2010; Lynch et al., 2015). Recent experimental and clinical evidence has suggested that abnormal activation of immune system may be involved in the process of cardiac function deterioration (Kawai, 1999; Lynch et al., 2017; Brayson et al., 2019). Exploring the mechanism of key immune cells, pathways, and molecules in the pathophysiological process of cardiomyopathy can help clarify the special role of the immune system in the maintenance and imbalance of cardiac function to some degree, so as to provide potential immunosuppressive targets for future immunotherapy.

By bioinformatics analyses, GO annotation and KEGG pathway enrichment analysis of the DEGs in merged dataset revealed that immune and inflammatory response and extracellular matrix remodeling play important roles in the pathogenesis of DCM, such as in myeloid leukocyte migration, cell chemotaxis, mononuclear cell migration, and extracellular matrix organization. Immune cell infiltration analysis indicated that the infiltration degrees of CD8^+^ T-cells and cDC were significantly enriched in DCM samples while Th1 cells and macrophages M1 were in healthy control tissues. According to PPI network analysis, ten hub genes were selected as potential biomarkers of DCM. Finally, three feature immune-related hub genes were identified as biomarkers by both correlation and logistic analyses with an excellent AUC score in the merged dataset of 40 DCM patients and 25 healthy controls. Our novel diagnosis model of DCM was constructed based on these three feature molecules and verified in another two datasets GSE5406 and GSE57338. The functions of the three biomarkers were summarized and displayed in Table 2.

**TABLE 2:** summary of three biomarkers.

Gene	General biological function	Function in cardiovascular disease
CC2	Chemoattractant for activated T lymphocytes and monocytes	Promotes the infiltration of mononuclear cells
	Primary activator for macrophages	Protects cardiomyocytes from death by autocrine and paracrine effects
CCL5	Chemoattractant for T lymphocytes, monocytes, eosinophils and NK cells	Promotes neutrophil and macrophage activator in myocardial infarction and myocarditis
TLR2	Recognizes pathogen-associated molecular patterns (PAMP) and damage-associated molecular patterns (DAMP)	Induces expression of inflammatory cytokines and chemokines, resulting in an invasion of inflammatory cells
		Protects heart in aged animals after transverse aortic constriction surgery

CCL2 (C-C motif chemokine ligand 2, also named as MCP-1) and CCL5 (C-C motif chemokine ligand 5, also named as RANTES) are two kinds of CC chemokines and play dual roles in inflammation and tissue repair. CCL2, the best studied chemokine, can be released by dendritic cells, monocytes, macrophages, smooth muscle cells and cardiomyocytes (Hanna and Frangogiannis, 2020). CCL2 is a potent chemoattractant for activated T lymphocytes and monocytes as well as a primary activator for macrophages (Rollins et al., 1988; Rollins, 1997). Previous studies suggested that CCL2 was upregulated in cardiac injury and was the key culprit for cardiac disease development and progression by promoting the infiltration of mononuclear cells (Hanna and Frangogiannis, 2020). CCL2 could also protect cardiomyocytes from cell death by its autocrine effect on cardiomyocytes and paracrine effect on endothelial cells, which stimulated angiogenesis (Tarzami et al., 2002; Hong et al., 2005; Tarzami et al., 2005). CCL5 is known to be a potent chemoattractant for T lymphocytes, monocytes, eosinophils, and natural killer cells (Jarrah and Tarzami, 2015). It is released by endothelial cells, smooth muscle cells, activated T-cells, macrophages, and so on upon inflammatory stimulus or infection (Jarrah and Tarzami, 2015). Although their function has been well studied on a systemic level, their role in cardiovascular disease, especially DCM, has not been fully elucidated.

TLR2 (Toll-like receptor 2) is a pattern-recognition receptor protein critical for the initiation of the innate immune system, which recognizes both pathogen-associated molecular patterns (PAMP) and damage-associated molecular patterns (DAMP) (Bryant et al., 2015). Besides TLR4, TLR2 is the next most abundant toll-like receptor in heart tissue (Mann, 2011). The binding of ligand to TLR2 induces expression of inflammatory cytokines and chemokines (e.g., IL-1β, TNF-α, CCL2), resulting in an invasion of macrophages and other inflammatory cells (Kawasaki and Kawai, 2014). Most of the previous reports suggested that TLR2 played a detrimental role upon pro-inflammation (Yu and Feng, 2018). However, other studies indicated a protective role of TLR2 in aged animals and mice after transverse aortic constriction surgery (Bualeong et al., 2016; Spurthi et al., 2018). In our study, we found that TLR2 was downregulated in DCM, which may lead to more sensitivity of DCM patients to injury stimulus, causing eventual cardiac dysfunction.

The novelties of our study were as follows. Firstly, we were the first to use bioinformatics analyses to investigate the molecular mechanism of DCM from the perspective of immunity and inflammation. Secondly, we identified that CCL2, CCL5 and TLR2 could be potential diagnostic biomarkers of DCM. Nonetheless, there are several limitations that should not be ignored. First, it cannot be determined whether there is a cause-and-effect relationship between gene expression differences and the pathophysiological mechanism of DCM or whether it was just compensatory change. Second, the study was a retrospective data analysis; thus, detailed clinical and prognostic profiles, such as the left ventricular eject fraction and the occurrence of adverse cardiovascular events in patients with DCM, were absent. This restricted the further exploration of the key genes about their clinical features and outcomes. Finally, our study was based on bioinformatics analyses of transcriptomic data of public datasets, which may be inconsistent with actual situation. Further clinical trials are needed to test our findings by bioinformatics analyses.

## Conclusion

By bioinformatics analyses of public transcriptional data, CCL2, CCL5, and TLR2 were identified as potential biomarkers of DCM from the perspective of immune cell infiltration combined with logistic regression. More importantly, a diagnostic model of DCM based on these three feature genes was developed, which brings a new aspect to the current understanding of the pathogenesis in DCM and may serve as interesting targets for future in-depth studies.

## Data Availability

Publicly available datasets were analyzed in this study. This data can be found here: GSE1145; GSE21610; GSE29819; GSE5406; GSE57338.

## Supplementary Material

Supplementary Figure S1 Venn diagrams depict the DEGs across two different integration methods. **(A)** Venn diagram of up-regulated DEGs. **(B)** Venn diagram of down-regulated DEGs. RRA: robust rank aggregation algorithm.

Supplementary Figure S2 Nine highly interconnected modules recognized by MCODE in DEGs.
