# Supplementary figures and images for: Identification and Verification of Feature Biomarkers Associated With Immune Cells in Dilated Cardiomyopathy by Bioinformatics Analysis
Source: Front Genet. 2022 May 12;13:874544. doi: 10.3389/fgene.2022.874544 (PMC9133742; doi:10.3389/fgene.2022.874544)

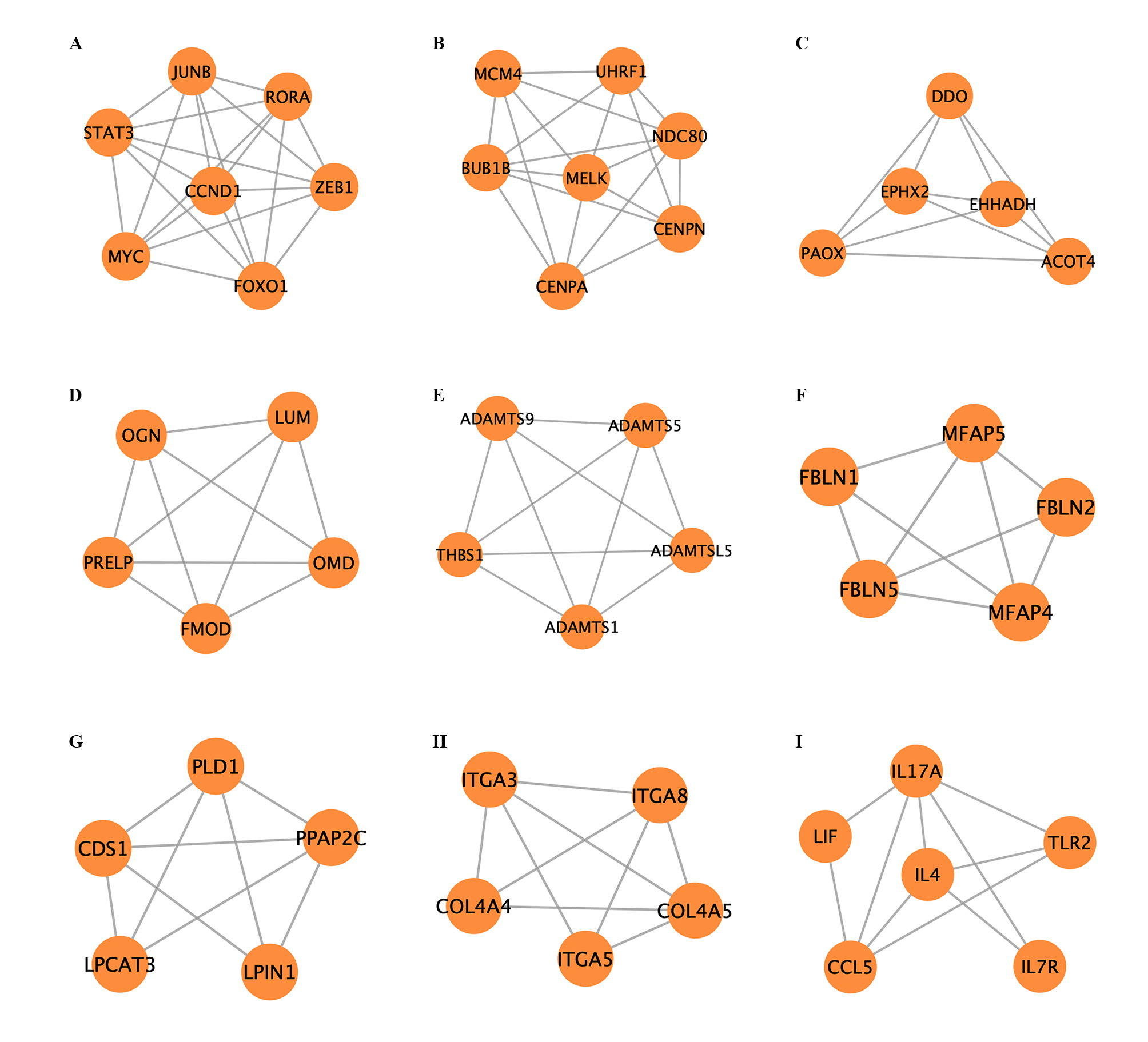

Supplement: Supplementary file 2 [file Image2.TIF]

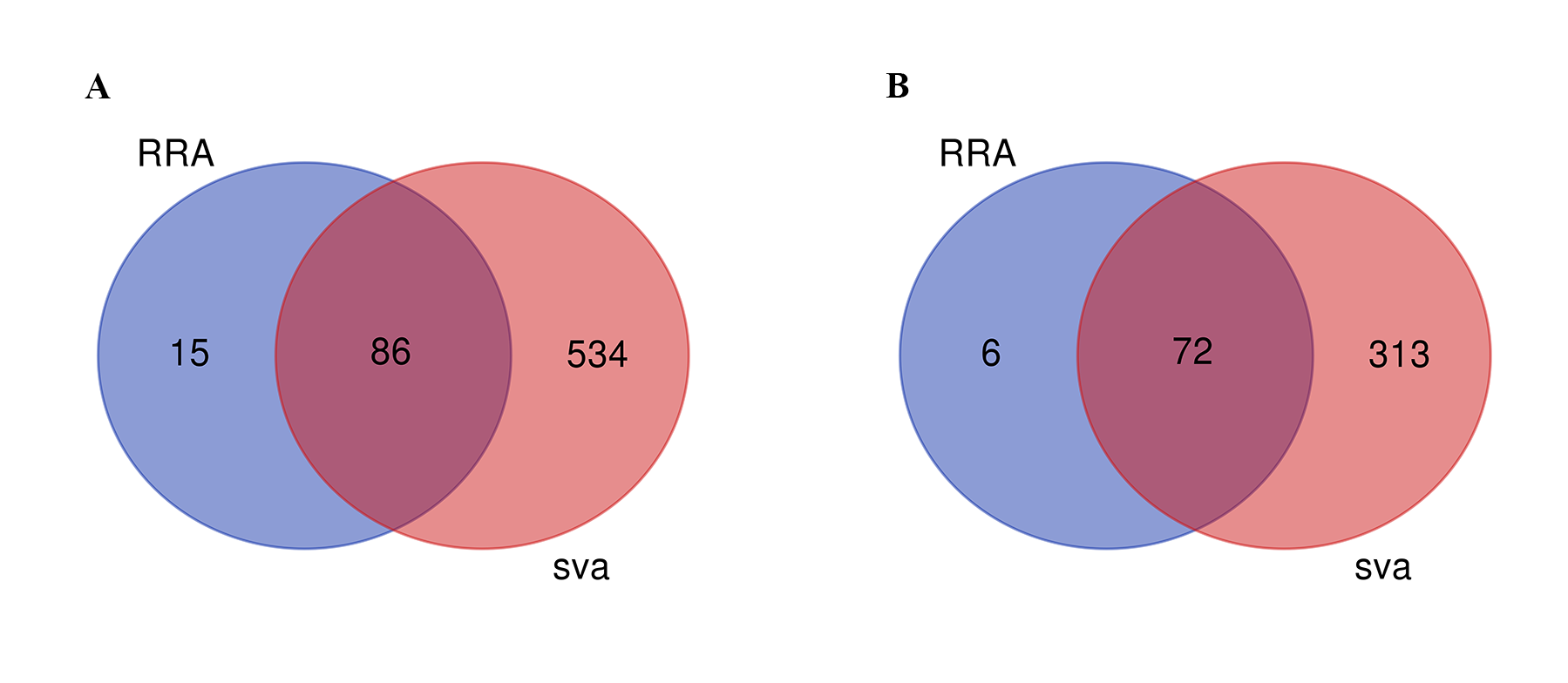

Supplement: Supplementary file 3 [file Image1.TIF]
